# Clinical Performance and Future Potential of Magnetic Resonance Thermometry in Hyperthermia

**DOI:** 10.3390/cancers13010031

**Published:** 2020-12-24

**Authors:** Theresa V. Feddersen, Juan A. Hernandez-Tamames, Martine Franckena, Gerard C. van Rhoon, Margarethus M. Paulides

**Affiliations:** 1 Department of Radiotherapy, Erasmus MC Cancer Institute, University Medical Center Rotterdam, 3015GD Rotterdam, The Netherlands; m.franckena@erasmusmc.nl (M.F.); g.c.vanrhoon@erasmusmc.nl (G.C.v.R.); m.m.paulides@tue.nl (M.M.P.); 2 Department of Radiology and Nuclear Medicine, Erasmus MC Cancer Institute, University Medical Center Rotterdam, 3015GD Rotterdam, The Netherlands; j.hernandeztamames@erasmusmc.nl; 3 Department of Applied Radiation and Isotopes, Reactor Institute Delft, Delft University of Technology, 2629JB Delft, The Netherlands; 4 Electromagnetics for Care & Cure Research Lab, Center for Care and Cure Technologies Eindhoven (C3Te), Department of Electrical Engineering, Eindhoven University of Technology, 5600MB Eindhoven, The Netherlands

**Keywords:** thermometry, thermal therapy, temperature mapping, magnetic resonance imaging, MRT, hyperthermia

## Abstract

**Simple Summary:**

Hyperthermia is a treatment for cancer patients, which consists of heating the body to 43 °C. The temperature during treatment is usually measured by placing temperature probes intraluminal or invasively. The only clinically used option to measure temperature distributions non-invasively and in 3D is by MR thermometry (MRT). However, in order to be able to replace conventional temperature probes, MRT needs to become more reliable. In this review paper, we propose standardized performance thresholds for MRT, based on our experience of treating nearly 4000 patients. We then review the literature to assess to what extent these requirements are already being met in the clinic today and identify common problems. Lastly, using pre-clinical results in the literature, we assess where the biggest potential is to solve the problems identified. We hope that by standardizing MRT parameters as well as highlighting current and promising developments, progress in the field will be accelerated.

**Abstract:**

Hyperthermia treatments in the clinic rely on accurate temperature measurements to guide treatments and evaluate clinical outcome. Currently, magnetic resonance thermometry (MRT) is the only clinical option to non-invasively measure 3D temperature distributions. In this review, we evaluate the status quo and emerging approaches in this evolving technology for replacing conventional dosimetry based on intraluminal or invasively placed probes. First, we define standardized MRT performance thresholds, aiming at facilitating transparency in this field when comparing MR temperature mapping performance for the various scenarios that hyperthermia is currently applied in the clinic. This is based upon our clinical experience of treating nearly 4000 patients with superficial and deep hyperthermia. Second, we perform a systematic literature review, assessing MRT performance in (I) clinical and (II) pre-clinical papers. From (I) we identify the current clinical status of MRT, including the problems faced and from (II) we extract promising new techniques with the potential to accelerate progress. From (I) we found that the basic requirements for MRT during hyperthermia in the clinic are largely met for regions without motion, for example extremities. In more challenging regions (abdomen and thorax), progress has been stagnating after the clinical introduction of MRT-guided hyperthermia over 20 years ago. One clear difficulty for advancement is that performance is not or not uniformly reported, but also that studies often omit important details regarding their approach. Motion was found to be the common main issue hindering accurate MRT. Based on (II), we reported and highlighted promising developments to tackle the issues resulting from motion (directly or indirectly), including new developments as well as optimization of already existing strategies. Combined, these may have the potential to facilitate improvement in MRT in the form of more stable and reliable measurements via better stability and accuracy.

## 1. Introduction

**Figure 1:**
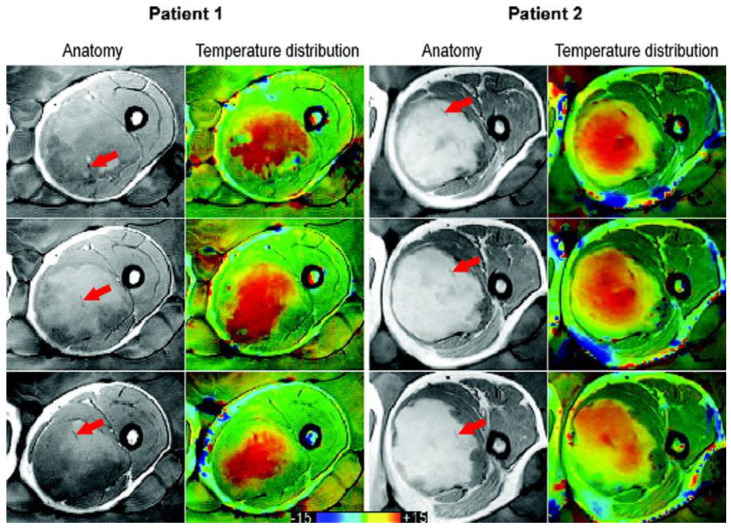
Taken from Gellermann et al. [21] and re-printed with permission from John Wiley and Sons. Example of anatomy with thermal mapping catheters (red arrows) for two patients with corresponding MR temperature distributions for three different slices. The images were acquired with a T1-weighted gradient-echo sequence. (The arrows were superimposed on the original image for clarity.)

Hyperthermia (39–43 °C) has been successful as a cancer treatment due to several beneficial effects on tissue, such as enhancing the efficacy of radiotherapy and chemotherapy [1,2]. The hallmarks of hyperthermia have been identified and are comprehensively presented by Issels et al. [3]. Due to these benefits, paired with no major side effects, hyperthermia has established itself in the clinic for many tumor sites [4,5,6,7]. Dose–effect studies show a positive association between thermal dose parameters and clinical outcome, which implies that real-time temperature dosimetry is essential [8,9,10,11,12,13,14]. Temperature during treatment is traditionally monitored by probes inside catheters that are placed inside lumina or pierced into tissue. These provide information at a limited number of points and may be difficult or unfeasible to place, or associated with complications [15,16]. Magnetic resonance thermometry (MRT) can provide a real-time 3D temperature map in a non-invasive way (Figure 1) and hence has the potential to make hyperthermia safer for the patient. Visualizing what is heated and to what extent is a necessary first step to be able to not only control hot spots in normal tissue and adapt to cold spots in tumor tissue, but also provide the means to perform a repeatable measurement, as well as to investigate the true optimum temperature for maximizing clinical outcome. MRT has been shown to correlate with pathological response in soft-tissue sarcomas of prospectively registered patients [17]. Despite this potential, MRT thus far has failed to establish itself as the standard temperature measurement method in hyperthermia treatments. Given the continued reported progress in the pre-clinical setting, we hypothesize that a major cause of this stagnation is the unclear validation status, as well as the non-standardized way of reporting pre-clinical performance. There is currently no overview of the clinical status quo of MRT in hyperthermia, and promising technologies are difficult to spot in the jungle of performance indicators. Further, the substantial financial investment will be overcome once the full contribution of MRT to hyperthermia quality is convincingly shown.

There have been many successful attempts to review the field. Rieke et al. [18] gives an overview of the different magnetic properties that can be exploited to obtain MRT. The importance of accuracy and stability of thermometry measurement are stressed, and acquisition and reconstruction methods that reduce motion artefacts are highlighted. Winter et al. [19] is expanding on those challenges faced, also supplying possible solutions. In addition to the hurdles, the implicit nature of the requirements for adequate MR temperature mapping during hyperthermia treatments complicates this quest. Different MRT techniques have different drawbacks and are thus suitable for different purposes of application. One example is the proton resonance frequency shift (PRFS), which is most frequently used to measure temperature due to its linear variability with temperature and, with the exception of fat, tissue independence. As the investigated shifts are very small, they are not easily able to deal with physiological changes, hence accurate temperature measurements are hampered by changes in the microenvironment of the tissue, for example in flow, oxygen levels, perfusion and magnetic properties of the blood. Lüdemann et al. [20] compared MRT techniques and their achievable accuracies. Despite these excellent reviews in the field, there has not been a comprehensive analysis of the validation status and a ranking of the pre-clinical work based on a clear set of performance indicators.

For patients to benefit from MRT in hyperthermia treatments, it needs to become reliable so that the invasive probes are no longer needed. Our objective is to identify how MRT can be improved to a point where the added value is appreciated in the clinic, leading to a more widespread use. In order to aid this development, we will firstly define minimum requirements for a successful treatment, creating a benchmark for more uniform reporting and clear comparisons across studies. Secondly, after a systematic literature search, clinical data will be used to assess to what extent the MRT performance metrics obtained satisfy these requirements. This will be used to identify areas of insufficiency, but also areas of overlap and common concerns. Finally, we will use pre-clinical data from the literature search to identify new techniques, which address those common concerns. By highlighting these ‘most promising to advance the field’ publications, we hope to emphasize the direction for future research and thus accelerate progress further.

## 2. Minimum Recommended Clinical MRT Performance

There are a lot of performance measures that can be evaluated and reported on, which in turn depend on many different acquisition settings. To clarify the situation, we introduce the most important acquisition parameters and state which MRT performance measures are vital to report on and define what minimum values we consider acceptable, based on the group’s expertise in nearly 4000 clinical (superficial and deep) hyperthermia treatments [6,9,22]. Our aim is to create a clear list of requirements of what is needed from a clinical MR guided hyperthermia treatment perspective. The focus is on MRT for mild and moderate hyperthermia (39–43 °C) only, hence excluding ablative temperatures. The latter has been the aim for most techniques, since MR guided thermal ablation has a much wider use. Compared to ablation, temperature changes in mild and moderate hyperthermia are slow (approximately 10–30 min to reach the target temperature), target regions are generally large, and the temperature changes from baseline are low (2–8 °C). Consequently, the desired temperature mapping performances are also different: although spatial resolution may be lower, measurement accuracy and stability (temporal temperature precision) must be high and robustness against confounders much better.

The minimum acquisition parameters we recommend for successful MRT are reported in Table 1 and the minimum MRT performances are shown in Table 2.

Considering the large areas of heating in hyperthermia and consequently low thermal gradients, we consider a reasonable minimum spatial and temporal resolution to be 125 mm^3^ (for instance 5 × 5 × 5 mm^3^). A higher spatial resolution may be required to achieve acceptable accuracy, by avoiding partial volume effects in regions with many small and contrasting tissues.

For this recommendation, we also considered the current spatial resolution that is achieved with invasive thermometry. In general, the distance between measuring points along a thermometry catheter track is 1–2 cm. The distance between thermometry catheters is much larger still, in the range of 5–10 cm. Additionally, the MRT resolution should be considered with respect to the resolution of our ability to steer the energy distribution. At this moment, the focus of the 100 MHz RF-deep heating has a diameter of 7–14 cm. For the Hypercollar3d operating at 434 MHz, this is 3 cm. Finally, when utilizing hyperthermia treatment planning for deep as well as head and neck treatments, the CT images used for planning are acquired with a slice width of 5 mm and a resolution of 0.98 mm in both x and y [23]. It is also worth considering that a higher resolution in hyperthermia treatment planning comes at the cost of increased intricacy and treatment time [24].

For deep heating, the clinical objective is to achieve a temperature increase between 0.5 and 2 °C per 5 min. If it is lower (<0.5 °C), the power is increased in order to speed it up; if it is higher (>2 °C), the power is reduced to slow it down. Because of these relatively slow heating times and the resulting high time constant of thermal washout, the minimum temporal resolution should be 20 s. This recommended minimum of the temporal resolution concerns the minimum acceptable time from a clinical perspective, and faster scanning may be required in regions of motion to achieve acceptable accuracy. Another reason to speed up the acquisition may be when the averaging of temperature data is required to achieve the minimum MRT performance, as stated in Table 2.

Regarding the important performance measures, the first mentioned in Table 2 is bias, measured as the mean error (ME), which is defined by Walther et al. [25] as:

(1) $$\mathrm{ME}=\frac{1}{n}\overset{n}{\underset{j=1}{\sum }}(E_{j}-A)$$

This is the difference between the MRT measurement (*E_j_*) and another temperature measurement that is considered true (*A*) over all measured time points (*n*). This reference *A*, i.e., the gold standard, can be a set of invasive temperature probes, or another MRT map originating from a well-established sequence. It is important to have a reliable and repeatable MRT readout, without a systematic over- or underestimation of temperature, translating to a low bias in measurements. Curto et al. made a comparison of the currently worldwide installed five RF-MR hybrid systems in anthropomorphic phantoms, showing with a mean error as low as 0.13 °C can be achieved with current systems in ‘ideal condition’ pre-clinical settings [26]. In light of the best resolution available, we consider a ME of ≤|0.5 °C| to be appropriate.

The following two measures, defined in Table 2, are spatial and temporal temperature precision. The spatial temperature standard deviation (SD) reflects the variability in the region of temperature evaluation, consisting of a ROI. Spatial temperature SD of the ROI evaluated should be ≤0.5 °C in order to guarantee that the noise present is not too large and there are no large temperature gradients within the heated region; in other words, the heated region is sufficiently uniform. Temporal temperature SD assesses the variability of the spatial mean temperature in a ROI across all time points and indicates the repeatability and stability of the measurement. Considering treatment times are long, but keeping in mind the importance of staying in the target temperature zone, the temporal temperature precision should not exceed 0.5 °C (after drift correction) for a 90 min thermometry measurement. Both the spatial and temporal temperature SD are influenced by the size and location of the ROI chosen. This, in turn, is highly dependent on the MRT region imaged, as areas with poor uniformity (for example near tissue/air boundaries) need to be avoided for sufficient accuracy of the measurement. Due to this needed flexibility, no recommendation on size and location of the ROI will be stated. In order to fulfil the minimum requirement of the temperature precision defined above, the ROI should be chosen with care in a region as uniform as possible. The measures of temperature precision are only valuable when the ROI is kept constant throughout the measured time points.

The final performance measure that is vital to report on is the accuracy of the MRT measurement. Accuracy, as stated by Walther et al. [25], can either be presented as the mean squared error (MSE), the root mean squared error (RMSE) or the mean absolute error (MAE). We consider the MAE the best one for our application since it is less sensitive for outliers and easy to interpret:

(2) $$\mathrm{MAE}=\frac{1}{n}\overset{n}{\underset{j=1}{\sum }}\left|E_{j}-A\right|$$

where *E_j_* is the MRT measurement, *A* is another temperature measurement that is considered true and n is the number of all measured time points. Given the importance of keeping to the right heating range for the desired physiological changes in the tumor tissue, we think it should be ≤1 °C.

## 3. Methods

### 3.1. Literature Search

In order to ensure that all papers published using MRT in hyperthermia treatments will be included, a logical search string was defined including a hyperthermia term, a magnetic resonance thermometry term, and excluding ablation in a major term. The search strings used for the different databases are provided in Appendix A. We searched the databases for papers published from inception of the databases until 24 November 2020. Details on the number of results obtained from the respective data bases are presented in Table A1.

Using the method from Wichor et al. [27], all papers were screened by title and abstracts for relevancy to our topic. At this stage, papers were excluded if they were not published in English, if they were not research articles or if the topic was not related to MRT in hyperthermia. Our definition of the combination of mild and moderate hyperthermia includes treatments with the heating goal between 39 and 45 °C. We acknowledge that in some cases tumor temperatures can be higher than the target temperature, thus papers up to 47 °C were considered relevant.

**Figure 2:**
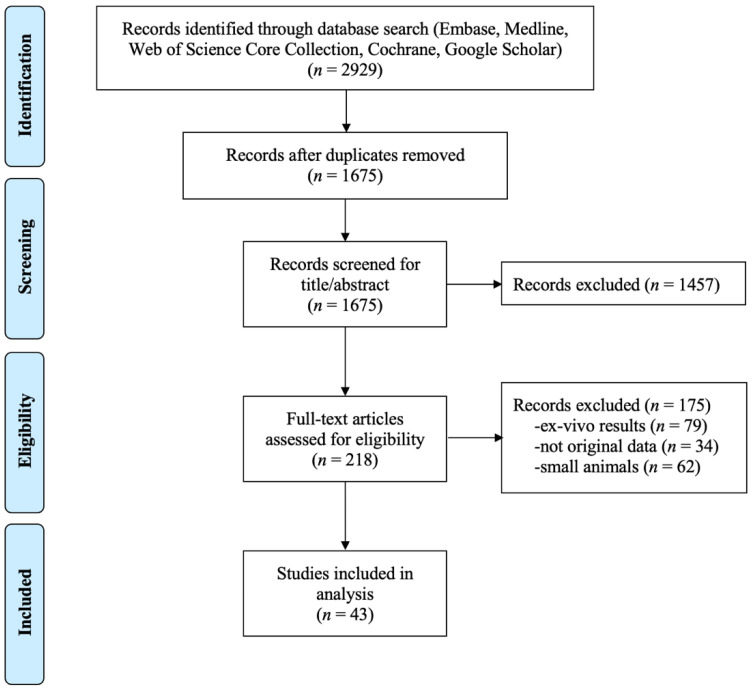
PRISMA flow chart.

The resulting 218 relevant papers were then assessed for eligibility, using the following exclusion criteria: (#1) ex-vivo results, (#2) not original data, and (#3) small animals. Ex vivo results excluded studies on simulation or phantoms, which we considered too far from the final intended use of the clinic to be included in this review. No original data excluded reviews and studies using already published data as reference. Small animals were considered to be anything smaller than a dog. These studies were excluded because we deem these data not predictive for humans due to the different motion profile (e.g., faster heart rate) and their smaller size. Additionally, the equipment used is specially made and non-clinical, lowering the ease of translation into the clinic. Large animal studies without heating were also not included. After this eligibility assessment, 43 papers remain to be included in the systematic analysis. A PRISMA flow chart of the exclusion process is shown in Figure 2.

### 3.2. Categories and Classification

The studies included were then categorized into patients with treatment intent and pre-clinical groups (with no hyperthermia treatment intent). Peller et al. [28] included treated and non-treated patients and thus was allocated to both groups. Clinical studies included 10 studies. Pre-clinical studies consisted of 35 studies: 26 papers with human subjects and 12 studies including large animals. In early volunteer studies, heating and cooling were sometimes applied to the volunteers without therapeutic intend. These studies were also considered pre-clinical.

The relevant papers were read in detail and relevant data were extracted into Microsoft Excel tables. The information, such as first author and year of publication, is the one obtained from the EndNote library. Other study data considered relevant were: hyperthermia treatment approach, imaging setup, MRT performance and the exclusion of data. Pre-clinical papers were also grouped and ranked based on their main aim and achieved improvement to identify promising techniques. Large animals, volunteers and non-heated patients are easier to image than treated patients, making it more likely for their MRT data to be artefact free. Large animals are typically sedated and mechanically ventilated during treatment, which reduces their breathing and makes it more predictable, and also lowers their blood perfusion. Muscle relaxant and bowel movement suppressants are also administered, minimizing any other avoidable motion. Volunteers have the advantage of no initial stress from illness and, when there is no heat applied, no additional stress during the treatment. Non-treated patients also lack the additional stress of treatment. Except for these differences, both large animals and volunteers have similar confounders such as size, motion profile and they generally use the same equipment for heating as well as imaging. Thus we consider these pre-clinical studies predictive for the reproducibility in patients during treatment.

## 4. MRT Performance in Clinical Studies

### 4.1. Status

MRT in hyperthermia is predominantly used for extremities (67%) and some studies investigated it in the pelvis (33%). This trend can be explained by the absence of motion and resulting artefacts in extremities. Data of ongoing research in our group show that achieving successful MRT in the pelvic area is much harder than in more static regions of the body. The average maximum temperature achieved during the hyperthermia treatments was 43.8 °C, which is well within the target treatment temperature range, and the treatment time varied from 30 to 90 min. All studies applied hyperthermia using radiofrequency (RF) electromagnetic waves. The most popular system is the BSD2000/3D/MR, which incorporates the twelve channel Sigma Eye applicator.

The imaging setup for the 10 clinical studies is presented in Table 3. The published MRT in hyperthermia clinical experience is limited to very few centers (Duke, Tubingen, Berlin, Munich). Hence there is a challenge on translating their high degree of specific experience to other centers. Additionally, it is difficult to define a benchmark due to the limited amount of data published.

The imaging coil used by most was the body coil, so when this information was absent, the body coil was assumed. MRT was based on the proton resonance frequency shift (PRFS), except in Peller et al. [28], who used T1. This is not surprising, as PRFS varies linearly with temperature over an adequate range and is near independent of tissue type [29]. Gradient Recalled Echo (GRE) sequences were generally used (Table 3), and all sequences acquired 2D MRT maps. Peller et al. [28] was the only study which used a 0.2 Tesla MRI instead of 1.5 T. The frequency of MRT acquisition varied from continuous to every 20 min. Studies that reported values for spatial and temporal resolutions within our recommended minimum of 125 mm^3^ and 20 s are shaded in green in Table 3. Most studies manage to satisfy the minimum requirements, as defined in Table 1 and Table 2.

Methods used to improve the thermometry quality were:

▪ Increasing the number of excitations (NEX) > 1 (number of times each k-space line is read) [30,31].
▪ Applying flow compensation [32].
▪ Including modelling of blood perfusion [33].
▪ Using background field removal algorithms to correct for motion-induced susceptibility artifacts [34].
▪ Only selecting evaluable volumes or treatments—all but one study (discussed in “Exclusion” section below).

Table 3 also shows the MRT performance reported in clinical papers. Values that meet our minimum requirements are shaded in green. Of the metrics that are reported, 6/9 of studies (67%) satisfy one or more of our minimum requirements.

Unsoeld et al. [17] shows the correlation of measured temperature with clinical outcome. Whilst this study investigates the true goal of the treatment, this study could have contributed more to the field if it had also reported bias, temperature SD and accuracy. This would have helped to understand the required treatment quality and the relationship between thermal dose and treatment outcome. A similar line of thought applies to the study of Wu et al. [34], which gives accounts of TNR improvement from their investigated correction method, but neglects to quantify these. Table 3 demonstrates that few performance metrics are reported, which makes it difficult to compare the status of MRT between different studies. Additionally, definitions of parameters are often lacking, leading to the need for educated guesses.

### 4.2. Exclusion of Data

Comparing these indicative performance metrics listed above comes with limitations. Often even the ROIs considered within the same study at different time points are not constant. Additionally, certain numbers of time points were often excluded from the evaluation—usually due to image artefacts that produce noisy thermometry maps. This decrease in the number of thermometry maps adds selection bias to the performances reported. In Table 4, we present what data were excluded post-acquisition and the reason why the authors excluded the data. If exclusion was not explicitly mentioned, we assumed that all MRT data acquired were also included in the analysis.

As is shown in Table 4, only one study included all of the acquired MRT data. This apparent need to exclude data underlines the need for MRT to become more reliable in regions of motion before it can replace invasive temperature probes. Information on the total study sizes also provides objective information on the practicality of using MRT. The limited number of publications on clinical use of MRT is highlighted and confirms that experience is very local (and presumably the conclusion on the feasibility of MRT is biased by the positive attitude of the researchers). All of the above clearly demonstrates that MRT is still in a developing phase and there exists a substantial need to make major improvements to expand to broader use of the technology.

### 4.3. Pre-Clinical Status—How Does It Compare?

**Figure 3:**
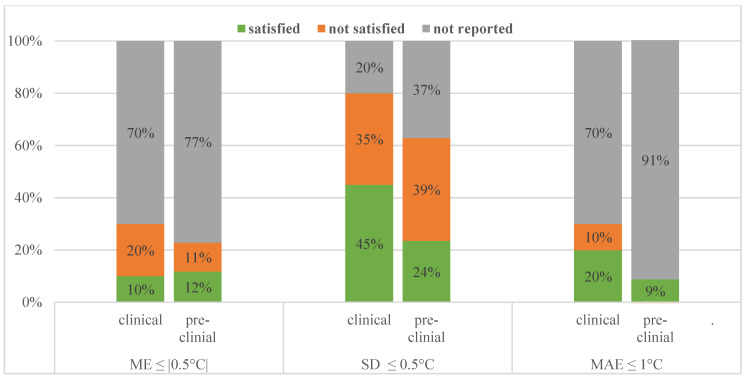
Performance values reported and how many of those satisfied our requirements for clinical and pre-clinical studies.

Comparing their imaging setup, pre-clinical studies are very similar to clinical ones. The sequences used and MRT methods used were more varied, but just like the patient studies investigated, the spatial resolution was met in all studies and temporal resolution requirement was met in 29/35 studies. Regarding MRT performance metrics about half of the pre-clinical studies achieved our minimum requirements. This is illustrated and contrasted to the clinical performance in Figure 3.

Considering exclusion of data post-acquisition, five pre-clinical studies (two with large animal and three with human subjects) excluded some, which is significantly less than the clinical studies investigated. This most likely can be linked back to the subjects making measurement conditions less challenging, as mentioned above.

### 4.4. New Techniques and Their Improvement

Table 5 presents the main techniques and methods investigated in the pre-clinical studies and their found improvement over standard methods. From this information, we have identified common main aims (last column).

When looking at the improvements mentioned over the benchmark methods (column 4 of Table 5), there was only one study that did not find an improvement in their investigated techniques (Mei et al. [52]). This demonstrates the importance and success of pre-clinical work.

The most promising techniques from Table 5 are the 13 studies satisfying our MRT performance criterion, these are highlighted in green. It can be seen that in recent years more studies have satisfied these minimum requirements. A total of 8 out of those 13 studies are feasibility or comparison studies (marked in grey in Table 5), which can be grouped into having investigated:

1. Hardware: MR-HIFU for different treatment location applications [45,47]
2. Thermometry method: MR spectroscopy to measure absolute temperature [64]
3. Sequences and parameters for MRT [43,56,57,70]
4. Performance of MRT at different anatomical sites [71]

The remaining 5/13 pre-clinical studies satisfying our MRT performance criteria and not involving feasibility or comparisons can be grouped by common main aims or problems to solve:

1. B0 drift: correction and stabilization strategies [37,60], navigator echoes [48];
2. B0 changes due to motion: breath hold [38], navigator echoes [48];
3. Image gaps due to motion: breath hold [38];
4. Acceleration: reconstruction method [50].

It needs to be highlighted that with the exception of Bing et al. [38], these studies have investigated only one subject, so their potential needs to be validated on a larger scale. Despite great potential, the possible limitations of the techniques mentioned above when transferred to patients in a clinical environment must be mentioned. Breath hold may not always be a viable option for the clinic. Some patients (for example young children) may not be able to hold their breath effectively, or may be sedated during the treatment. Additionally, the total treatment time will lengthen, as the patient needs periods of normal breathing to recover. Navigators to correct for B0 changes induced by breathing may only be valuable in areas with no motion in the treated area, as well as very regular breathing patterns. Similarly, B0 drift corrections may only be valuable in areas with no motion or motion induced changes present.

It can be seen from the investigated studies that groups working on MRT advances are generally different groups than those working on patients with a treatment objective. The consequence is that solutions are presented with very limited ability to be transferred to clinical practice in RF-hyperthermia MRT. This is highlighted by the fact that no progress has been made in MRT for pelvic RF-hyperthermia in the past two decades: the body coil is still used for imaging and PRFS is used with no real solution for correction of external and internal movement, for instance by passing air.

However, the technological advances in recent years are promising in many ways. Multi-coil integrated hyperthermia systems are becoming available and provide much better SNR than the commonly used body coil [55,72]. At the same time, multi-coil acquisition also offers acceleration of MRT by techniques such as, for example, parallel imaging or compressed sensing [73]. This faster acquisition enables better temperature monitoring, especially in regions with motion. Additionally, the computational power available now is much bigger and cheaper compared to only a few years ago, which makes more complicated reconstruction method and correction strategies feasible [34]. Last but not least, new sequences and approaches are being developed to increase MRT performance and explore the possibility to perform MRT in more difficult regions. In early years, researchers broadly investigated different methods of MRT, but PRFS quickly crystalized as the method of choice for hyperthermia treatments for reasons aforementioned. It is only now, that other methods are being considered again. Hybrid approaches of PRFS/T1 MRT are just one method on the horizon that enables temperature mapping in fatty regions [74], which PRFS alone would be unable to detect.

Considering all these innovations, the present conditions are favorable to push MRT to the next level and hopefully, in the near future, have the powers to elevate it from a research modality to clinical practice.

## 5. Conclusions

Standardized reporting of parameters used and performances obtained in MRT is important. Hence we defined a minimum benchmark of important performance metrics including bias, spatial and temporal temperature precision as well as accuracy; these should be within ≤|0.5 °C|, ≤0.5 °C, ≤0.5 °C and ≤1 °C, respectively.

When systematically assessing the literature, we can conclude that MRT performance in hyperthermia is already achieving most of these requirements for extremities but not yet in regions with more motion present. Motion as well as the B0 changes, as a direct or indirect consequence, emerged as the main problem of accurate and reliable MR temperature measurement.

Various techniques satisfying our performance requirements are already available at the pre-clinical stage addressing these problems. Most promising common solution proposals can be divided into either new approaches or optimizations. New approaches include hardware or software being developed; propitious optimizations include correction and stabilization strategies, navigator echoes, breath hold and various reconstruction methods. We anticipate that highlighting these promising pre-clinical advancements will accelerate the progress of MRT.

## Figures and Tables

**Table 1:** Minimum acquisition parameters, such that successful MRT in hyperthermia can be achieved.

Parameter	Definition	Minimum
Spatial resolution	In-plane resolution times slice width (2D) or through-plane resolution (3D)	125 mm^3^
Temporal resolution	Time needed to acquire one MRT slice	20 s

**Table 2:** Minimum performance metrics for successful MRT in hyperthermia treatments.

Measure	Metric	Definition	Minimum
Bias	Mean error (ME)	$\mathrm{ME}=\frac{1}{n}\overset{n}{\underset{j=1}{\sum }}(E_{j}-A)$	≤|0.5 °C|
Spatial temperature precision	Spatial temperature standard deviation (SD)	${\mathrm{SD}}^{2}=\frac{1}{n}\overset{n}{\underset{j=1}{\sum }}{\left(E_{j}-\bar{E}\right)}^{2}$	≤0.5 °C
Temporal temperature precision	Temporal temperature standard deviation (SD)	Variability at different time points over 90 min	≤0.5 °C
Accuracy	Mean absolute error (MAE)	$\mathrm{MAE}=\frac{1}{n}\overset{n}{\underset{j=1}{\sum }}\left|E_{j}-A\right|$	≤1 °C

**Table 3:** Imaging setup for clinical papers.

Author (year)	Body Part	Sequence	Spatial Res (mm^3^)	Temporal Res (s)	ME (°C)	Spatial Temperature Precision (°C)	Temporal Temperature Precision (°C)	MAE (°C)
Carter (1998) [35]	E	GRE	8.8	-	-	0.50	-	-
Craciunescu (2009) [31]	E	GRE	27.4	10	-	0.52	-	0.74
Craciunescu (2001) [33] ^†^	E	GRE	11.9	-	-	0.49~	-	-
	E	GRE	13.7	-	-	0.56~	-	-
		EPI	156.0	1				
Dadakova (2015) [32]	E,P	EPI	67.6	1.08	−0.04	0.55	-	0.40
		GRE	152.1	3.12				
Gellermann (2005) [36]	P	GRE	146.8	3.12	-	2.10	-	1.50
Gellermann (2006) [21]	E,P	GRE	146.8	3.12	1.10	0.70	-	-
Peller (2002) [28] *^,^^	E,P	GRE	96.1	64	-	0.10	-	-
Stauffer (2009) [30]	E	GRE	21.1	15	0.85	-	-	-
Unsoeld (2020) [17]	E	not stated	-	-	-	0.21	-	-
Wu (2020) [34]	P	GRE	152.1	3.32	-	-	-	-

^†^ Supplied a 95% confidence interval of the MR temperature. * Used T1 instead of PRFS for calculating MR temperature maps. ^ Uses a 0.2 T instead of a 1.5 T MRI. ~ Standard error instead of SD. Body part imaged: E = extremities, P = pelvis. Metrics that are within our recommended minimum are shaded in green (spatial and temporal resolutions of 125 mm^3^ and 20 s, respectively).

**Table 4:** Exclusions of data from clinical studies post-acquisition.

Author	All Data included?	Size of Study	What Was Excluded?	Why?
Carter (1998) [35]	No	4 patients 5 treatments	Not stated	Artefacts
Craciunescu (2001) [33]	Yes	2 patients	-	-
Craciunescu (2009) [31]	No	10 patients 40 treatments	4 patients 12 treatments	Lack of MR information in HT treatments performed outside the MR scanner, image/motion artefacts, uncorrectable drift, impossibility to localize the fiber optic probes, missing/corrupted data files
Dadakova (2015) [32]	No	3 patients 20 treatments	1 patient 1 treatment	Susceptibility artefact in the ROI from air in rectum
Gellermann (2005) [36]	No	15 patients	Everything but 1 best session per patient	MR data sets incomplete and/or disturbed by technical reasons
Gellermann (2006) [21]	No	9 patients 30 treatments	15 treatments	Breakdown or malfunction of applicator, restlessness of the patient
Peller (2002) [28]	No	1 patient	“Data sets”	Artefacts
Stauffer (2009) [30]	No	10 patients	3 patients All except 12 treatments	Uncorrected field drift or inability to locate or correlate sensor positions or significant patient position shift early in treatment
Unsoeld (2020) [17]	No	24 patients	13 patients: 11 patients with abdominal and pelvic tumors; 1 patient with different time course of therapy; 1 patient without surgery	Breathing and intestinal motion artefacts in MRT data; pathological response is not comparable; lack of information on pathological response
Wu (2020) [34]	No	4 patients	2 patients	Bulk motion due to discomfort during treatment, ROI contained too much gas

**Table 5:** Pre-clinical studies by the year of publication; including technique/method investigated, improvement found (where applicable) and main aim of the study. Feasibility and comparison studies are presented in grey. Studies satisfying our performance criterion are highlighted in green.

Author	Year	Technique/Method Investigated	Improvement	Main Aim
Wu [34]	2020	Correction of motion-induced susceptibility artifacts	TNR improvement	B0 changes and image gaps due to motion, B0 drift
Ferrer [37]	2020	Different B0 drift corrections	IQR improved from 9.31 to 0.80 °C. ME improved from −4.30 to 0.33 °C	B0 drift
Bing [38]	2019	Forced breath-hold MR-HIFU	Accuracy and stability from 1.2 to 0.6 °C and from 1.4 to 0.8 °C	B0 changes and image gaps due to motion
Odeen [39]	2019	Different protocols for PRFS MRT for LITT	Factor 2 improvement in the temperature SD	Comparison
Tan [40]	2019	Motion compensation using principal component analysis and projection onto dipole fields	Reduces temperature SD from 3.02 to 0.86 °C	B0 changes and image gaps due to motion
Wu [41]	2019	Novel fast spin echo method	TNR efficiency improved by 25%	Feasibility
Zhu [42]	2019	Feasibility/safety of MRgHIFU	N/A	Feasibility
Jonathan [43]	2018	Proposed and validated a hybrid radial-EPI temperature mapping pulse sequence	Provides whole brain coverage, temperature SD was 48% higher than standard	Feasibility
Kothapalli [44]	2018	MRT performance at different anatomical sites	N/A	Comparison
Chu [45]	2016	Feasibility (safety + performance) MRgHIFU for rectal cancer	Precision and stability of temperature improved from 7.8 and 2.3 °C to 0.3 and 0.6 °C	Feasibility
Svedin [46]	2016	Correction of respiration artifact in 3D MRT using phase navigators	Temperature measurement improved by a factor of 2.1	B0 changes and image gaps due to motion
Tillander [47]	2016	Hyperthermia for deep-seated heating volumes using HIFU	N/A	Feasibility
Boulant [48]	2015	FID navigator to correct for B0 field and variations induced by breathing	Reduces the temperature SD of the data over the first 8 min from 0.2 to 0.05 °C	B0 drift and B0 changes due to motion
De Senneville [49]	2015	Approach for motion estimation of abdominal organs	Temperature SD improvement of 0.4 °C and reduction of artefacts by up to 3 °C	B0 changes and image gaps due to motion
Gaur [50]	2015	Reconstruction method to accelerate MRT	Achieves same temperature error at up to 32× acceleration factors	Acceleration
Marx [51]	2015	MASTER sequence	Temperature SD improvement from 1.21 to 0.82 °C	Feasibility
Mei [52]	2015	Different methods for B0 inhomogeneity correction	None	B0 drift
Pichardo [53]	2014	Multi-baseline MR-based thermometry	Reduced temperature SD from 25.2 to 2.4 °C	B0 changes due to motion
Shi [54]	2014	partial separability (PS) model and referenceless thermometry introduced	N/A	Feasibility
Minalga [55]	2013	Integrated multi-channel RF receive coil with MR-HIFU	163% SNR improvement averaged over all positions investigated	Feasibility
Ramsay [56]	2013	Segmented GRE-EPI technique	N/A	Feasibility
Kickhefel [57]	2010	Comparison of fast sequences	Stability improvement from 1.07 to 0.21 °C	Comparison
Wyatt [58]	2010	Correction of breathing-induced errors using multi-echo fitting methods	Temperature SD from 2.18 to 0.61 °C and bias from 3.17 to −1.26 °C	B0 changes and image gaps due to motion
Roujol [59]	2009	Reconstruction pipeline for adaptive TSENSE	Image latencies below 90 ms at frame rates up to 40 images/s	Acceleration
Wyatt [60]	2009	Different stabilization strategies	Improved error by up to 0.5 °C	B0 drift
Silcox [61]	2005	Ultrasonic heating to control transgene expression spatially using a minimally invasive approach	N/A	Feasibility
Sun [62]	2005	Adaptive controllers with MRT	N/A	Feasibility
Peller [28]	2002	Characterize T1 for thermometry	N/A	Feasibility
Il’yasov [63]	1998	RARE sequence for diffusion MRT	N/A	Feasibility
Corbett [64]	1997	1H MR spectroscopy to measure absolute brain temperature	N/A	Feasibility
MacFall [65]	1996	Chemical shift of water for MRT	N/A	Feasibility
De Poorter [66]	1995	PRF thermometry in vivo	N/A	Feasibility
MacFall [67]	1995	Rapid diffusion weighted EPI, being less sensitive to motion	Temperature SD from 1.5 to 0.9 °C	B0 changes due to motion
Young [68]	1994	Initial investigation of T1 dependence, D and perfusion	N/A	Feasibility
Hall [69]	1990	Investigation which MR parameter would be best for MRT in vivo	N/A	Comparison

## Abbreviations

FOV	Field of view
MAE	Mean absolute error
ME	Mean error
MR-HIFU	MR-high intensity focused ultrasound
MRT	Magnetic resonance thermometry
MSE	Mean squared error
NEX	Number of excitations
PRFS	Proton resonance frequency shift
RF	Radiofrequency
RMSE	Root mean squared error
ROI	Region of interest
SD	Standard deviation

## Appendix A. Detailed Search Strings

**Table A1:** Databases searched for research articles. In brackets is the date of inception of the database. The 2nd column shows the number of search results and the 3rd column the number of search results after removing duplicates.

DATABASE	Number of Results	Number of Results after Removing Duplicates
Embase (1971–)	1292	1345
Medline ALL Ovid (1946–)	723	113
Web of Science Core Collection (1975–)	693	55
Cochrane CENTRAL register of trials (1992–)	21	15
Google scholar	200	147
**Total**	**2929**	**1675**

Original search strings of the respective databases introduced in Figure 2.

### Appendix A.1. Embase.com (1971–) 1292

(‘hyperthermia’/de OR ‘thermotherapy’/exp OR ‘high intensity focused ultrasound’/exp OR (hyperthermi* OR thermotherap* OR ((therm* OR heat) NEAR/3 therap*) OR ((high-intensit*) NEAR/3 ultras*)):ab,ti) AND (‘magnetic resonance thermometry’/exp OR ‘MR-guided focused ultrasound’/exp OR ((‘nuclear magnetic resonance’/exp OR ‘nuclear magnetic resonance imaging’/exp) AND (‘thermometry’/de OR ‘temperature measurement’/de OR ‘thermometer’/de)) OR (((magnetic OR proton*) NEAR/3 (resonance) NEAR/6 (thermometr* OR thermogra* OR guid* OR controlled*)) OR ((MR OR mri OR nmr OR MRT OR Prf) NEAR/6 (thermometr* OR thermogra* OR guid* OR controlled*)) OR ((MR OR mri OR nmr OR MRT OR Prf) NEAR/6 (temperature* OR thermal*) NEAR/6 (mapping* OR map OR maps OR imag* OR measure* OR monitoring* OR dosing* OR dosage OR value* OR change*)) OR ((magnetic OR proton*) NEAR/3 (resonance) NEAR/6 (temperature* OR thermal*) NEAR/6 (mapping* OR map OR maps OR imag* OR measure* OR monitoring* OR dosing* OR dosage OR value* OR change*))):ab,ti) NOT (‘ablation therapy’/exp/mj OR (ablation*):ti) NOT ([conference abstract]/lim) AND [English]/lim.

### Appendix A.2. Medline ALL Ovid (1946–) 723

(Hyperthermia, Induced/ OR (hyperthermi* OR thermotherap* OR ((therm* OR heat) ADJ3 therap*) OR ((high-intensit*) ADJ3 ultras*)).ab,ti.) AND (((exp Magnetic Resonance Imaging/) AND (exp Thermometry/ OR Thermometers/)) OR (((magnetic OR proton*) ADJ3 (resonance) ADJ6 (thermometr* OR thermogra* OR guid* OR controlled*)) OR ((MR OR mri OR nmr OR MRT OR Prf) ADJ6 (thermometr* OR thermogra* OR guid* OR controlled*)) OR ((MR OR mri OR nmr OR MRT OR Prf) ADJ6 (temperature* OR thermal*) ADJ6 (mapping* OR map OR maps OR imag* OR measure* OR monitoring* OR dosing* OR dosage OR value* OR change*)) OR ((magnetic OR proton*) ADJ3 (resonance) ADJ6 (temperature* OR thermal*) ADJ6 (mapping* OR map OR maps OR imag* OR measure* OR monitoring* OR dosing* OR dosage OR value* OR change*))).ab,ti.) NOT (exp *Ablation Techniques/OR (ablation*).ti.) NOT (news OR congres* OR abstract* OR book* OR chapter* OR dissertation abstract*).pt. AND english.la.

### Appendix A.3. Web of Science Core Collection (1975–) 693

AB = (((hyperthermi* OR thermotherap* OR ((therm* OR heat) NEAR/2 therap*) OR ((high-intensit*) NEAR/2 ultras*))) AND ((((magnetic OR proton*) NEAR/2 (resonance) NEAR/5 (thermometr* OR thermogra* OR guid* OR controlled*)) OR ((MR OR mri OR nmr OR MRT OR Prf) NEAR/5 (thermometr* OR thermogra* OR guid* OR controlled*)) OR ((MR OR mri OR nmr OR MRT OR Prf) NEAR/5 (temperature* OR thermal*) NEAR/5 (mapping* OR map OR maps OR imag* OR measure* OR monitoring* OR dosing* OR dosage OR value* OR change*)) OR ((magnetic OR proton*) NEAR/2 (resonance) NEAR/5 (temperature* OR thermal*) NEAR/5 (mapping* OR map OR maps OR imag* OR measure* OR monitoring* OR dosing* OR dosage OR value* OR change*))))) NOT (TI = (ablation*)) AND DT = (article) AND LA = (english).

### Appendix A.4. Cochrane CENTRAL Register of Trials (1992–) 21

((hyperthermi* OR thermotherap* OR ((therm* OR heat) NEAR/3 therap*) OR ((high-intensit*) NEAR/3 ultras*)):ab,ti) AND ((((magnetic OR proton*) NEAR/3 (resonance) NEAR/6 (thermometr* OR thermogra* OR guid* OR controlled*)) OR ((MR OR mri OR nmr OR MRT OR Prf) NEAR/6 (thermometr* OR thermogra* OR guid* OR controlled*)) OR ((MR OR mri OR nmr OR MRT OR Prf) NEAR/6 (temperature* OR thermal*) NEAR/6 (mapping* OR map OR maps OR imag* OR measure* OR monitoring* OR dosing* OR dosage OR value* OR change*)) OR ((magnetic OR proton*) NEAR/3 (resonance) NEAR/6 (temperature* OR thermal*) NEAR/6 (mapping* OR map OR maps OR imag* OR measure* OR monitoring* OR dosing* OR dosage OR value* OR change*))):ab,ti) NOT ((ablation*):ti).

### Appendix A.5. Google Scholar 200

hyperthermia|thermotherapy “magnetic|proton resonance” thermometry|thermography|“temperature|thermal mapping|map|measurement|monitoring”-ablation.

## References

[1] Van den Tempel N., Horsman M.R., Kanaar R. (2016). Improving efficacy of hyperthermia in oncology by exploiting biological mecha-nisms. Int. J Hyperth..

[2] Datta N.R., Kok H.P., Crezee H., Gaipl U.S., Bodis S. (2020). Integrating Loco-Regional Hyperthermia Into the Current Oncology Practice: SWOT and TOWS Analyses. Front. Oncol..

[3] Issels R., Kampmann E., Kanaar R., Lindner L.H. (2016). Hallmarks of hyperthermia in driving the future of clinical hyperthermia as targeted therapy: Translation into clinical application. Int. J. Hyperth..

[4] Datta N., Ordóñez S.G., Gaipl U., Paulides M.M., Crezee J., Gellermann J., Marder D., Puric E., Bodis-Wollner I. (2015). Local hyperthermia combined with radiotherapy and-/or chemotherapy: Recent advances and promises for the future. Cancer Treat. Rev..

[5] Wust P., Hildebrandt B., Sreenivasa G., Rau B., Gellermann J., Riess H., Felix R., Schlag P.M. (2002). Hyperthermia in combined treatment of cancer. Lancet Oncol..

[6] Van der Zee J., González D.G., Rhoon G.C., Van Dijk J.D.P., Van Putten W.L.J., Hart A.A.M. (2000). Comparison of radiotherapy alone with radiotherapy plus hyperthermia in locally advanced pelvic tumours: A pro-spective, randomised, multicentre trial. Lancet.

[7] Issels R.D. (2008). Hyperthermia adds to chemotherapy. Eur. J. Cancer.

[8] Kroesen M., Mulder H.T., Van Holthe J.M., Aangeenbrug A.A., Mens J.W.M., Van Doorn H.C., Paulides M.M., Hoop E.O.-D., Vernhout R.M., Lutgens L.C. (2019). Confirmation of thermal dose as a predictor of local control in cervical carcinoma patients treated with state-of-the-art radiation therapy and hyperthermia. Radiother. Oncol..

[9] Franckena M., Fatehi D., De Bruijne M., Canters R.A.M., Van Norden Y., Mens J.W., Van Rhoon G.C., Van Der Zee J. (2009). Hyperthermia dose-effect relationship in 420 patients with cervical cancer treated with combined radiotherapy and hyperthermia. Eur. J. Cancer.

[10] Jones E.L., Oleson J.R., Prosnitz L.R., Samulski T.V., Vujaskovic Z., Yu D., Sanders L.L., Dewhirst M.W. (2005). Randomized Trial of Hyperthermia and Radiation for Superficial Tumors. J. Clin. Oncol..

[11] Fotopoulou C., Cho C.-H., Kraetschell R., Gellermann J., Wust P., Lichtenegger W., Sehouli J. (2010). Regional abdominal hyperthermia combined with systemic chemotherapy for the treatment of patients with ovari—An cancer relapse: Results of a pilot study. Int. J. Hyperth..

[12] Rau B., Wust P., Tilly W., Gellermann J., Harder C., Riess H., Budach V., Felix R., Schlag P.M. (2000). Preoperative radio-chemotherapy in locally advanced recurrent rectal cancer: Regional radiofrequency hyperthermia correlates with clinical parameters. Int. J. Radiat. Oncol. Biol. Phys..

[13] Kapp D.S., Cox R.S. (1995). Thermal treatment parameters are most predictive of outcome in patients with single tumor nodules per treat-ment field in recurrent adenocarcinoma of the breast. Int. J. Radiat. Oncol. Biol. Phys..

[14] Oleson J.R., Samulski T.V., Leopold K.A., Clegg S.T., Dewhirst M.W., Dodge R.K., George S.L. (1993). Sensitivity of hyperthermia trial outcomes to temperature and time: Implications for thermal goals of treatment. Int. J. Radiat. Oncol..

[15] Van Der Zee J., Peer-Valstar J.N., Rietveld P.J., De Graaf-Strukowska L., Van Rhoon G.C. (1998). Practical Limitations of Interstitial Thermometry During Deep Hyperthermia. Int. J. Radiat. Oncol..

[16] Sneed P.K., Dewhirst M.W., Samulski T., Blivin J., Prosnitz L.R. (1998). Should interstitial thermometry be used for deep hyperthermia?. Int. J. Radiat. Oncol. Biol. Phys..

[17] Unsoeld M., Lamprecht U., Traub F., Hermes B., Scharpf M.O., Potkrajcic V., Zips D., Paulsen F., Eckert F. (2020). MR Thermometry Data Correlate with Pathological Response for Soft Tissue Sarcoma of the Lower Extremity in a Single Center Analysis of Prospectively Registered Patients. Cancers.

[18] Rieke V., Pauly K.B. (2008). MR thermometry. J. Magn. Reson. Imaging.

[19] Winter L., Oberacker E., Paul K., Ji Y., Oezerdem C., Ghadjar P., Thieme A., Budach V., Wust P., Niendorf T. (2015). Magnetic resonance thermometry: Methodology, pitfalls and practical solutions. Int. J. Hyperth..

[20] Lüdemann L., Wlodarczyk W., Nadobny J., Weihrauch M., Gellermann J., Wust P. (2010). Non-invasive magnetic resonance thermography during regional hyperthermia. Int. J. Hyperth..

[21] Gellermann J., Hildebrandt B., Issels R., Ganter H., Wlodarczyk W., Budach V., Felix R., Tunn P.-U., Reichardt P., Wust P. (2006). Noninvasive magnetic resonance thermography of soft tissue sarcomas during regional hyperthermia: Correlation with response and direct thermometry. Cancer.

[22] Sherar M., Liu F.-F., Pintilie M., Levin W., Hunt J., Hill R., Hand J., Vernon C., Van Rhoon G., Van Der Zee J. (1997). Relationship between thermal dose and outcome in thermoradiotherapy treatments for superficial recurrences of breast cancer: Data from a phase III trial. Int. J. Radiat. Oncol..

[23] Canters R.A.M., Paulides M.M., Franckena M.F., Van Der Zee J., Van Rhoon G.C. (2012). Implementation of treatment planning in the routine clinical procedure of regional hyperthermia treatment of cervical cancer: An overview and the Rotterdam experience. Int. J. Hyperth..

[24] Kok H.P., Wust P., Stauffer P.R., Bardati F., Van Rhoon G.C., Crezee J. (2015). Current state of the art of regional hyperthermia treatment planning: A review. Radiat. Oncol..

[25] Walther B.A., Moore J.L. (2005). The concepts of bias, precision and accuracy, and their use in testing the performance of species richness estimators, with a literature review of estimator performance. Ecography.

[26] Curto S., Aklan B., Mulder H.T., Mils O., Schmidt M., Lamprecht U., Peller M., Wessalowski R., Lindner L.H., Fietkau R. (2019). Quantitative, Multi-institutional Evaluation of MR Thermometry Accuracy for Deep-Pelvic MR-Hyperthermia Systems Operating in Multi-vendor MR-systems Using a New Anthropomorphic Phantom. Cancers.

[27] Bramer W.M., Milic J., Mast F. (2017). Reviewing retrieved references for inclusion in systematic reviews using EndNote. J. Med. Libr. Assoc..

[28] Peller M., Reinl H.M., Weigel A., Meininger M., Issels R.D., Reiser M. (2002). T1 relaxation time at 0.2 Tesla for monitoring regional hyperthermia: Feasibility study in muscle and adipose tissue. Magn. Reson. Med..

[29] Yuan J., Panych L.P., McDannold N.J., Madore B., Mei C.-S. (2012). Towards fast and accurate temperature mapping with proton resonance frequency-based MR thermometry. Quant. Imaging Med. Surg..

[30] Stauffer P.R., Craciunescu O.I., Maccarini P.F., Wyatt C., Arunachalam K., Arabe O., Stakhursky V., Li Z., Soher B., MacFall J.R. (2009). Clinical utility of magnetic resonance thermal imaging (MRTI) for realtime guidance of deep hyperthermia. Proc. SPIE.

[31] Craciunescu O.I., Stauffer P.R., Soher B.J., Wyatt C.R., Arabe O., Maccarini P., Das S.K., Cheng K.-S., Wong T.Z., Jones E.L. (2009). Accuracy of real time noninvasive temperature measurements using magnetic resonance thermal imaging in patients treated for high grade extremity soft tissue sarcomas. Med. Phys..

[32] Dadakova T., Gellermann J., Voigt O., Korvink J.G., Pavlina J.M., Hennig J., Bock M. (2014). Fast PRF-based MR thermometry using double-echo EPI: In vivo comparison in a clinical hyperthermia setting. Magma Magn. Reson. Mater. Phys. Biol. Med..

[33] Craciunescu O.I., Das S.K., McCauley R.L., MacFall J.R., Samulski T.V. (2001). 3D numerical reconstruction of the hyperthermia induced temperature distribution in human sarcomas using DE-MRI measured tissue perfusion: Validation against non-invasive MR temperature measurements. Int. J. Hyperth..

[34] Wu M., Mulder H.T., Baron P., Coello E., Menzel M.I., Van Rhoon G.C., Haase A. (2020). Correction of motion-induced susceptibility artifacts and B 0 drift during proton resonance frequency shift-based MR thermometry in the pelvis with background field removal methods. Magn. Reson. Med..

[35] Carter D.L., MacFall J.R., Clegg S.T., Wan X., Prescott D.M., Charles H.C., Samulski T.V. (1998). Magnetic Resonance Thermometry during Hyperthermia for Human High-Grade Sarcoma. Int. J. Radiat. Oncol..

[36] Gellermann J., Wlodarczyk W., Hildebrandt B., Ganter H., Nicolau A., Rau B., Tilly W., Fähling H., Nadobny J., Felix R. (2005). Noninvasive Magnetic Resonance Thermography of Recurrent Rectal Carcinoma in a 1.5 Tesla Hybrid System. Cancer Res..

[37] Ferrer C.J., Bartels L.W., Van Der Velden T.A., Grüll H., Heijman E., Moonen C.T.W., Bos C. (2019). Field drift correction of proton resonance frequency shift temperature mapping with multichannel fast alternating nonselective free induction decay readouts. Magn. Reson. Med..

[38] Bing C., Cheng B., Staruch R.M., Nofiele J., Staruch M.W., Szczepanski D., Farrow-Gillespie A., Yang A., Laetsch T.W., Chopra R. (2019). Breath-hold MR-HIFU hyperthermia: Phantom and in vivo feasibility. Int. J. Hyperth..

[39] Odéen H., Parker D.L. (2019). Magnetic resonance thermometry and its biological applications—Physical principles and practical consider-ations. Prog. Nucl. Magn. Reson. Spectrosc..

[40] Tan J., Mougenot C., Pichardo S., Drake J.M., Waspe A.C. (2019). Motion compensation using principal component analysis and projection onto dipole fields for abdominal magnetic reso-nance thermometry. Magn. Reson. Med..

[41] Wu M., Mulder H.T., Zur Y., Lechner-Greite S., Menzel M.I., Paulides M.M., Van Rhoon G.C., Haase A. (2018). A phase-cycled temperature-sensitive fast spin echo sequence with conductivity bias correction for monitoring of mild RF hyperthermia with PRFS. Magma Magn. Reson. Mater. Phys. Biol. Med..

[42] Zhu L., Lam D., Pacia C.P., Gach H.M., Partanen A., Talcott M.R., Greco S.C., Zoberi I., Hallahan D.E., Chen H. (2019). Feasibility and safety assessment of magnetic resonance-guided high-intensity focused ultrasound (MRgHIFU)-mediated mild hyperthermia in pelvic targets evaluated using an in vivo porcine model. Int. J. Hyperth..

[43] Jonathan S.V., Grissom W.A. (2018). Volumetric MRI thermometry using a three-dimensional stack-of-stars echo-planar imaging pulse sequence. Magn. Reson. Med..

[44] Kothapalli S.V.V.N. (2018). Evaluation and selection of anatomic sites for magnetic resonance imaging-guided mild hyperthermia thera-py: A healthy volunteer study. Int. J. Hyperth..

[45] Chu W. (2016). Magnetic resonance-guided high-intensity focused ultrasound hyperthermia for recurrent rectal cancer: MR thermome-try evaluation and preclinical validation. Int. J. Radiat. Oncol. Biol. Phys..

[46] Svedin B.T., Payne A., Parker D.L. (2016). Respiration artifact correction in three-dimensional proton resonance frequency MR ther-mometry using phase navigators. Magn. Reson. Med..

[47] Tillander M., Hokland S., Koskela J., Dam H., Andersen N.P., Ylihautala M., Pedersen M., Tanderup K., Köhler M.O. (2016). High intensity focused ultrasound induced in vivo large volume hyperthermia under 3D MRI temperature control. Med. Phys..

[48] Boulant N. (2015). FID navigator-based MR thermometry method to monitor small temperature changes in the brain of ventilated animals. NMR Biomed..

[49] De Senneville B.D., El Hamidi A., Moonen C. (2015). A Direct PCA-Based Approach for Real-Time Description of Physiological Organ Deformations. IEEE Trans. Med. Imaging.

[50] Gaur P., Grissom W.A. (2015). Accelerated MRI thermometry by direct estimation of temperature from undersampled k-space data. Magn. Reson. Med..

[51] Marx M., Plata J., Pauly K.B. (2015). Toward Volumetric MR Thermometry with the MASTER Sequence. IEEE Trans. Med. Imaging.

[52] Mei C.-S., Chu R., Hoge W.S., Panych L.P., Madore B. (2014). Accurate field mapping in the presence of B0 inhomogeneities, applied to MR thermometry. Magn. Reson. Med..

[53] Pichardo S., Kohler M., Lee J., Hynnyen K. (2014). In vivo optimisation study for multi-baseline MR-based thermometry in the context of hyperthermia using MR-guided high intensity focused ultrasound for head and neck applications. Int. J. Hyperth..

[54] Shi C., Xie G., Song Y., Zou C., Liu X., Zhou S. (2013). Referenceless PRFS MR Thermometry Using Partial Separability Model. Appl. Magn. Reson..

[55] Minalga E., Payne A., Merrill R., Todd N., Vijayakumar S., Kholmovski E., Parker D.L., Hadley J.R. (2013). An 11-channel radio frequency phased array coil for magnetic resonance guided high-intensity focused ultrasound of the breast. Magn. Reson. Med..

[56] Ramsay E., Mougenot C., Köhler M., Bronskill M., Klotz L., Haider M.A., Chopra R. (2013). MR thermometry in the human prostate gland at 3.0T for transurethral ultrasound therapy. J. Magn. Reson. Imaging.

[57] Kickhefel A., Roland J., Weiss C., Schick F. (2010). Accuracy of real-time MR temperature mapping in the brain: A comparison of fast sequences. Phys. Med..

[58] Wyatt C., Soher B.J., MacFall J.R. (2010). Correction of breathing-induced errors in magnetic resonance thermometry of hyperthermia using multiecho field fitting techniques. Med. Phys..

[59] Roujol S., De Senneville B.D., Vahala E., Sørensen T.S., Moonen C., Ries M. (2009). Online real-time reconstruction of adaptive TSENSE with commodity CPU/GPU hardware. Magn. Reson. Med..

[60] Wyatt C., Soher B., Maccarini P.F., Charles H.C., Stauffer P.R., MacFall J. (2009). Hyperthermia MRI temperature measurement: Evaluation of measurement stabilisation strategies for extremity and breast tumours. Int. J. Hyperth..

[61] Silcox C.E., Smith R.C., King R., McDannold N., Bromley P., Walsh K., Hynynen K. (2005). MRI-guided ultrasonic heating allows spatial control of exogenous luciferase in canine prostate. Ultrasound Med. Biol..

[62] Sun L., Collins C.M., Schiano J.L., Smith M.B., Smith N.B. (2005). Adaptive Real-Time Closed-Loop Temperature Control for Ultrasound Hyperthermia Using Magnetic Resonance Ther-mometry. Concepts Magn. Reson. Part B Magn. Reson. Eng..

[63] Il’yasov K.A., Hennig J. (1998). Single-shot diffusion-weighted RARE sequence: Application for temperature monitoring during hyperthermia session. J. Magn. Reson. Imaging.

[64] Corbett R., Laptook A., Weatherall P. (1997). Noninvasive Measurements of Human Brain Temperature Using Volume-Localized Proton Magnetic Resonance Spectroscopy. Br. J. Pharmacol..

[65] MacFall J.R., Prescott D.M., Charles H.C., Samulski T.V. (1996). 1 H MRI phase thermometry in vivo in canine brain, muscle, and tumor tissue. Med. Phys..

[66] De Poorter J., De Wagter C., De Deene Y., Thomsen C., Ståhlberg F., Achten E. (1995). Noninvasive MRI Thermometry with the Proton Resonance Frequency (PRF) Method:In Vivo Results in Human Muscle. Magn. Reson. Med..

[67] MacFall J., Prescott D.M., Fullar E., Samulski T.V. (1995). Temperature dependence of canine brain tissue diffusion coefficient measured in vivo with magnetic resonance echo-planar imaging. Int. J. Hyperth..

[68] Young I.R., Hand J.W., Oatridge A., Prior M.V. (1994). Modeling and observation of temperature changes in vivo using MRI. Magn. Reson. Med..

[69] Hall A.S., Prior M.V., Hand J.W., Young I.R., Dickinson R.J. (1990). Observation by MR Imaging of In Vivo Temperature Changes Induced by Radio Frequency Hyperthermia. J. Comput. Assist. Tomogr..

[70] Odéen H., Parker D.L. (2019). Improved MR thermometry for laser interstitial thermotherapy. Lasers Surg. Med..

[71] Kothapalli S.V.V.N., Partanen A., Zhu L., Altman M.B., Gach H.M., Hallahan D.E., Chen H. (2018). A convenient, reliable, and fast acoustic pressure field measurement method for magnetic resonance-guided high-intensity focused ultrasound systems with phased array transducers. J. Ther. Ultrasound.

[72] Paulides M.M., Drizdal T., Van Rhoon G.C., Yeo D. (2018). Novel Applicator Design for MR Guided RF Hyperthermia in Head and Neck Cancers: Heating Performance and RF Coupling.

[73] Nakagawa Y., Kokuryo D., Kaihara T., Fujii N., Kumamoto E. (2019). Image reconstruction method with compressed sensing for high-speed MR temperature measurement of abdominal organs. IEEE Conf. Proc. Eng. Med. Biol. Soc..

[74] Todd N., Diakite M., Payne A., Parker D.L. (2014). In vivo evaluation of multi-echo hybrid PRF/T1 approach for temperature monitoring during breast MR-guided focused ultrasound surgery treatments. Magn. Reson. Med..

